# Supplementary material for: Observation of the Antimicrobial Activities of Two Actinomycetes in the Harvester Ant Messor orientalis
Source: Insects. 2022 Jul 31;13(8):691. doi: 10.3390/insects13080691 (PMC9408877; doi:10.3390/insects13080691)
Supplement: Supplementary file 1 [file insects-13-00691-s001.zip › insects-1822753-supplementary.pdf]

Observation of the Antimicrobial Activities of Two Actinomycetes in the Harvester Ant *Messor orientalis*

Yiyang Wu [1,2], Yaxuan Liu [3], Jinyong Yu [4], Yijuan Xu [1,*] and Siqi Chen [1,*]

[1] Guangdong Laboratory for Lingnan Modern Agriculture, Red Imported Fire Ant Research Center, South China Agricultural University, Guangzhou 510642, China; yiyang_hongling@163.com
[2] Sendelta International Academy, Shenzhen 518000, China
[3] Department of Material Science and Engineering, College of Engineering, Carnegie Mellon University, Pittsburgh, PA 15213-2683, USA; yaxuanl2@andrew.cmu.edu
[4] College of Agronomy and Biotechnology, Hebei Normal University of Science & Technology, Qinhuangdao 066600, China; yujing_75211@163.com
* Correspondence: Author, Red Imported Fire Ant Research Center, South China Agricultural University, Guangzhou 510642, China; xuyijuan@scau.edu.cn (Y.X.); chensq@stu.scau.edu.cn (S.C.)

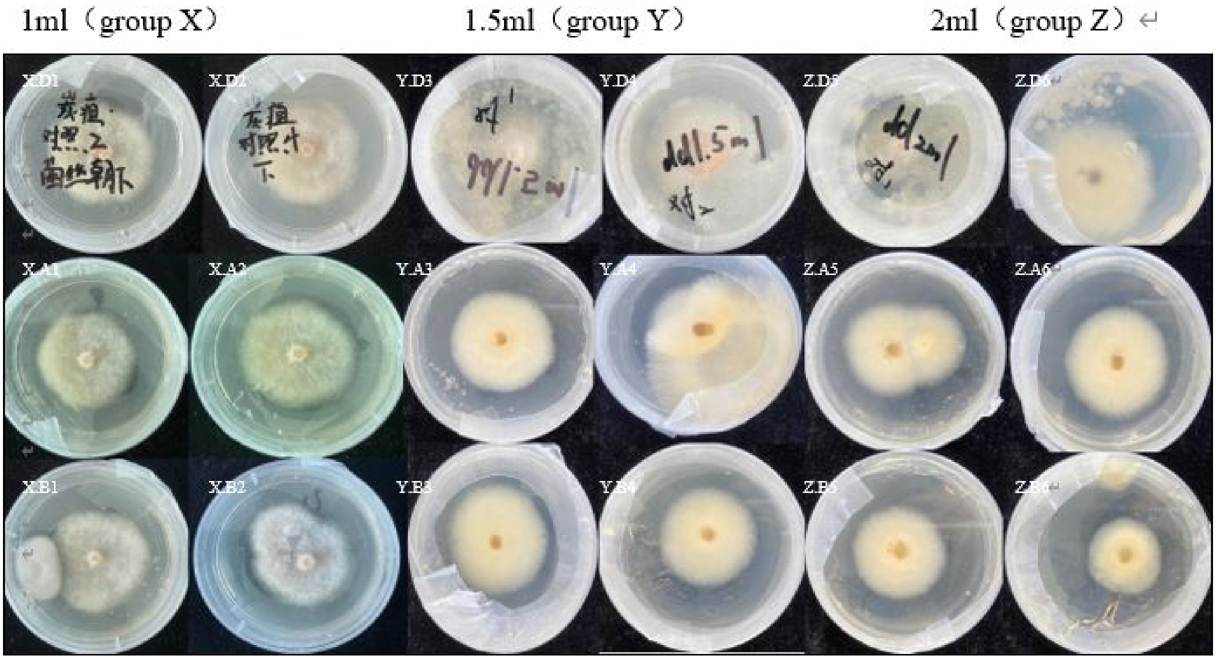

Figure S1. Growth of *Colletotrichum siamense* on various inhibitory media. (A) *Brachybacterium phenoliresistens* MO, (B) *Microbacterium* sp., (D) control.

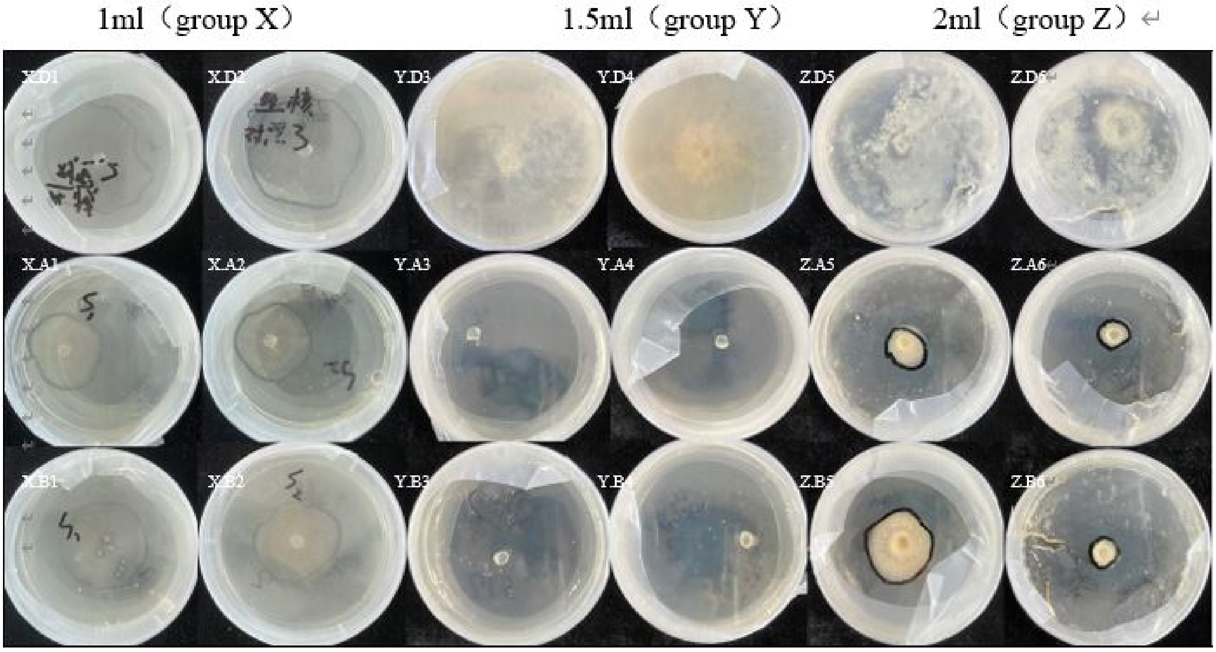

Figure S2. Growth of *Rhizoctonia solani* on various inhibitory media. (A) *Brachybacterium phenoliresistens* MO, (B) *Microbacterium* sp., (D) control.

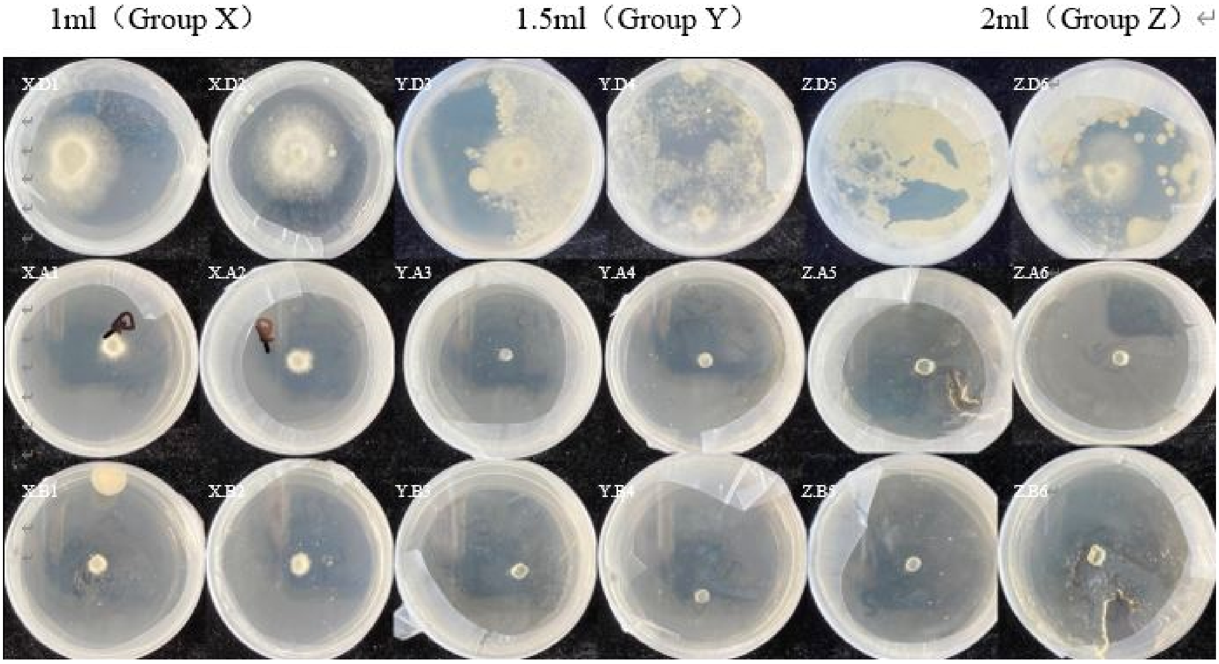

Figure S3. Growth of *Peronophthora litchii* on various inhibitory media. (A) *Brachybacterium phenoliresistens* MO, (B) *Microbacterium* sp., (D) control.
